# Estimating and Comparing Dam Deformation Using Classical and GNSS Techniques

**DOI:** 10.3390/s18030756

**Published:** 2018-03-02

**Authors:** Riccardo Barzaghi, Noemi Emanuela Cazzaniga, Carlo Iapige De Gaetani, Livio Pinto, Vincenza Tornatore

**Affiliations:** Politecnico di Milano, Department of Civil and Environmental Engineering (DICA)—Geomatics and Geodesy Section, Piazza Leonardo da Vinci 32, 20133 Milan, Italy; riccardo.barzaghi@polimi.it (R.B.); noemi.cazzaniga@polimi.it (N.E.C.); carloiapige.degaetani@polimi.it (C.I.D.G.); livio.pinto@polimi.it (L.P.)

**Keywords:** dam, monitoring, GNSS, predictive modeling, collimators, pendulum, displacement detection, time series

## Abstract

Global Navigation Satellite Systems (GNSS) receivers are nowadays commonly used in monitoring applications, e.g., in estimating crustal and infrastructure displacements. This is basically due to the recent improvements in GNSS instruments and methodologies that allow high-precision positioning, 24 h availability and semiautomatic data processing. In this paper, GNSS-estimated displacements on a dam structure have been analyzed and compared with pendulum data. This study has been carried out for the Eleonora D’Arborea (Cantoniera) dam, which is in Sardinia. Time series of pendulum and GNSS over a time span of 2.5 years have been aligned so as to be comparable. Analytical models fitting these time series have been estimated and compared. Those models were able to properly fit pendulum data and GNSS data, with standard deviation of residuals smaller than one millimeter. These encouraging results led to the conclusion that GNSS technique can be profitably applied to dam monitoring allowing a denser description, both in space and time, of the dam displacements than the one based on pendulum observations.

## 1. Introduction

Monitoring is fundamental for characterizing the structural behavior of dams and for identifying potential damages. In fact, an accurate definition of the structural response is required for preserving the safety of such large structures and the surrounding areas.

Dams are typically monitored on a regular basis with different types of instruments. For instance, they can be installed with piezometers, total stress cells, settlement devices, triaxial deformation tubes, inclinometers and extensometers, plumb lines, uplift pressure cells and laser alignment systems.

During the last decade other monitoring approaches have been considered, based on both contact and remote sensors that show mutually complementary characteristics. A review on different techniques (terrestrial and space) used for dam monitoring is presented by [1]. An example is the use of terrestrial laser scanners [2,3] that can describe the actual shape of the dam with a very high level of detail and accuracy. Nevertheless, the measurements, rather expensive, are performed only with low frequency (typically some months), so the data do not allow detection of the structure oscillations. Synthetic Aperture Radar (SAR) techniques from satellites can be adopted, as well [4,5]. They are very useful for non-instrumented dams; in fact the frequency of the surveying campaigns is generally higher than the classical topographical ones (satellites can survey the same area with intervals of the order of some days) and they observe the surrounding area, too. Limiting factors can be the fact that the displacement is estimated just along a single direction or the irregular satellite availability (see for example the time series realized by [6]), even if recently a method for increasing the temporal sampling in some situations was proposed by [7]. Also, terrestrial SAR has been proposed [8]. In this case, measurement campaigns will probably have the same frequency as the classical topographical surveys.

Another option is to install GNSS (Global Navigation Satellite Systems) devices on the dam. Through them, the movements of single points on the dam crest can be monitored. Thanks to the huge amount of data which can be gathered (in terms of temporal frequency) and the possibility of multiple deployments, through suitable processing methods an accurate description of the structure behavior can be obtained, leading to near real-time alert systems. The idea of using GNSS for dam monitoring already arose in 1988, see [9], but the first realization was in 1995 on the Pacoima dam [10]. This first experiment gathered quarter-daily estimates for two years, demonstrating the feasibility of the approach, despite the limitations of the GNSS algorithms available at that time. After this successful first experiment, many dams were monitored (also) with GNSS during the last years, sometimes with automatic configurations [11,12,13,14,15,16,17,18]. Generally, oscillations and displacements obtained from GPS measurements are used to define or validate structural models of the monitored dam. In few cases the GNSS results are directly compared with the displacements detected by other instruments, as, for instance, gravity-based plumb lines [11], coordimeters [19], pendulums [20], or angular collimators [21]. Nevertheless, all the problems related to the different reference systems of the various devices are not explicitly addressed. This is probably because data gathered by different sensors are never directly merged to obtain a unique estimate of the movements. A single roto-translation has been presented as the method for transforming the GNSS coordinates to a local reference frame at the dam crest [16], but alignment of other sensors is not considered. This aspect has been addressed in this work.

In this paper, analyses are carried out with the aim of assessing the position precision that can be acquired using GNSS. This is done by comparing GNSS-observed displacements with those coming from pendulums that are placed in correspondence of GNSS instruments. In Section 2, the Cantoniera dam is described together with the pendulum and GNSS devices that are deployed there, a short description on collimator data is also given. In Section 3, the method for aligning the pendulum and the GNSS data is presented and the analytical models fitting pendulum and GNSS observation are estimated. Comments on the obtained results and possible future applications of GNSS techniques for dam monitoring are given in Section 4.

## 2. Cantoniera Dam Monitoring System 

**Figure 1:**
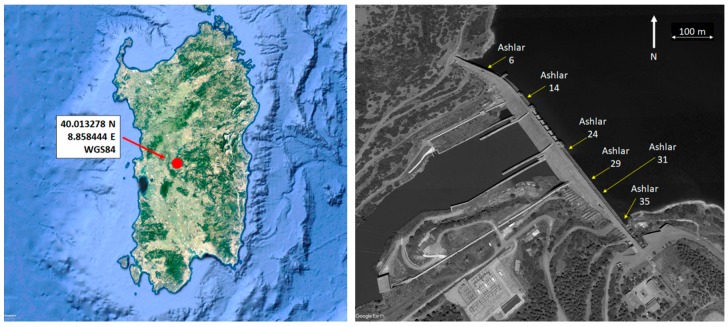
Geographical position of the Cantoniera dam in Sardinia (Italy), on the **left**: dam aerial view with indicated ashlar positions, on the **right** (credits Map data ©2015 Google).

**Figure 2:**
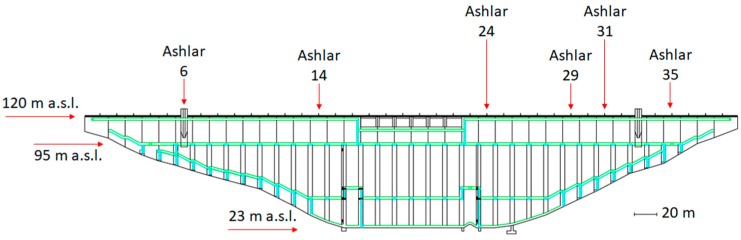
Front section of the Cantoniera dam and ashlar deployment.

This paper tackles the deformation study of a dam located in Sardinia (Italy), called Eleonora D’Arborea or Cantoniera dam. Waters gathered from Tirso river give rise to the Omodeo lake (see Figure 1), that is one of the largest artificial basins of Europe having a water full capacity of 792.84 × 10^6^ m^3^. This huge reservoir supplies drinking water, but it is also used for irrigation and for hydroelectric energy generation. Due to hydrogeological characteristics of the area, Cantoniera dam has an original shape. It is a hollow gravity dam with 38 deployed ashlars (each 15 m long and 4 m wide, see Figure 2). The dam is 100 m high and 582 m long, and the structure is monitored by 90 extensometers, 122 mono-axial and 4 tri-axial joint-meters. In addition, almost each ashlar has targets for collimation measures and 14 of them are equipped with pendulum chambers, having installed two optical instruments (direct and reverse pendulums with both 0.01 mm accuracy). Recently, also a GNSS monitoring system has been installed.

The dam is managed by ENte Acque Sardegna (ENAS) that has contributed to this study providing monitoring data and ancillary information about the dam. In particular, regarding the monitoring data, they refer to three different systems, based on collimators, pendulums and GNSS receivers. 

Collimation observations, in a number equal to 158, have been collected over the period January 2004–April 2017 on a monthly basis. In the frame of dam monitoring, collimation technique allows measuring the relative displacement of targets, located at the top of the dam, along a unique direction geometrically determined and parallel to the upstream-downstream direction. 

Regarding pendulum data, 107 observations have been acquired, roughly once a month from July 2006 to April 2017. Displacement measurements provided by the direct and inverted pendulums have been combined, giving a unique observation. This combination provides the displacement between the anchorage point of the inverted pendulum (fixed and with height depending on the depth of the foundation of the ashlar on which it is installed) and the fasten point of the direct pendulum (free to move together with the ashlar on which it is installed at fixed height above the sea level). In the case of the Cantoniera dam, pendulum measurements refer to displacements occurring at 95 m a.s.l. (above sea level) and they have been linearly propagated up to the top of the dam at 120 m a.s.l. Every pendulum measures the displacements along two orthogonal directions, transversal and longitudinal to the dam crest. 

As already mentioned above, recently, a GNSS monitoring system has been installed, too. The GNSS network has been materialized through several double-frequency GNSS antennas along the top of the dam (monitoring points) and in the nearby stable areas (reference points). The system is fully automatized and remotely managed by specific software. Three permanent GNSS stations are located on fixed benchmarks outside the dam but preliminary tests showed that only one of them remained stable during the experiment. Therefore, just this one has been used as master with respect to six rover stations deployed in correspondence of ashlars 6, 14, 24, 29, 31 and 35 (see Figure 2).

**Figure 3:**
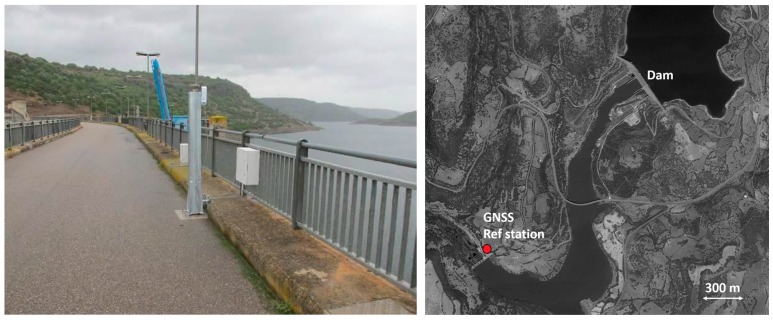
One of the GNSS antenna monitoring the dam and the position of the reference station.

Firstly, three GNSS receivers (Leica GMX902) with AX1202GG antennas, were installed on the ashlars number 14, 24 and 35. Their data became available at the beginning of August 2013. The remaining three ashlars were equipped with GNSS receivers during the subsequent year; observations were provided starting from October 2014. Observation frequency is 1 Hz. Positions of the monitoring points were computed on a daily basis with respect to the reference station installed in a stable area about 1.8 km away from the dam body (see Figure 3). GNSS data have been processed with the Leica GNSS Spider software, using a cut-off angle of 15° and broadcast ephemerides, by evaluating static post-processed phase double differences. In such a way, 3D daily coordinates of each monitoring point have been estimated in the WGS84 reference frame.

Since the ashlars n. 24, 29 and 31 have been monitored by all the three methodologies on overlapping periods, the displacement analyses carried out in this work is focused on them. Then daily GNSS solutions estimated on these ashlars constitute a time series of about 840 values in the period from October 2014 to April 2017, while for pendulums and collimators, 30 observations are available in the same period. The investigations on these time series will be presented in the next section. 

## 3. Time Series Comparison and Modeling

In order to properly compare the data collected with the different techniques, it must be considered that, in general, GNSS, pendulums and collimation monitoring systems are installed at different positions. Thus, the observed displacements reflect the dam deformation in different ways. Therefore, in order to compare them, proper reference systems must be defined, and data must be reduced to them accordingly. In this paper, it was decided to consider only the GNSS and the pendulum derived observations, which have been made comparable following an approach that is based on the observed data. Collimator data have been only considered in a general discussion in the conclusions, being quite sparse in time and measuring displacements just along one single direction, i.e., the cross-crest one.

### 3.1. Defining the Reference Frames for Comparing the GNSS and the Pendulum Horizontal Displacements

For each GNSS station point, a local level reference system has been defined. The origin has been put equal to the average latitude (lat.), longitude (lon.) and ellipsoidal height (h.) of the coordinates estimated in the period from 10 December 2014 to 9 December 2015, the North direction (N) is tangent to the meridian, northward, and the East direction (E) is tangent to the parallel, eastward. Then, the GNSS-estimated World Geodetic System 1984 (WGS84) geographic coordinates (lat., lon., h) have been roto-translated in the (N, E, Up) local coordinates defined on each GNSS station point.

**Figure 4:**
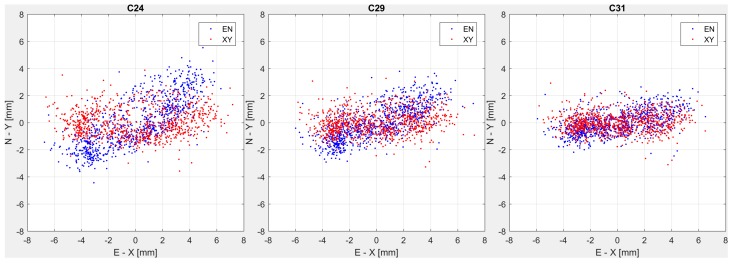
Displacements estimated on ashlars 24 (**left**), 29 (**center**) and 31 (**right**) by GNSS in the East-North (blue dots) and in the X-Y (red dots) reference systems in the period 10 December 2014 to 9 December 2015.

If these (N, E) coordinates are plotted for the three GNSS stations that are in correspondence with the three pendulum devices, placed at ashlars 24, 29 and 31 respectively, they result in clouds of points as it is represented in Figure 4 (blue dots). The three point clouds are roughly distributed as an ellipse having the two semi-axes with different magnitude and orientation, depending on the considered ashlar. Considering the same observation period, the studied part of the crest (left hydraulic side of the dam) does not move homogeneously. The displacements increase from the eastern dam edge to the center, in fact points are more dispersed for ashlar 24 that is the most central, while the opposite behavior is observed in ashlar 31, close to the constrained edge of the dam.

Then, for each ashlar, the E-N frame has been rotated to an X-Y orthogonal frame having the X axis aligned to the direction of maximum displacement as derived by the observations. The rotation angle between the two reference systems has been evaluated by estimating via Least Square Adjustment (LSA) the angular coefficient “a” of the straight-line model North = a∙East. This has been done using one year of data during the same time span used above. The resulting angles α_i_ = arctan(a_i_) (i = 1, 2, 3) are 28.0°, 18.4° and 9.3° for ashlars 24, 29 and 31 (with standard deviation of 1.3°, 0.9° and 0.8°) respectively.

Finally, for each GNSS station, the GNSS-observed displacements in the East-North frame have been rotated to the corresponding (X,Y) frame (see Figure 4, red dots). It must be stressed that, by applying this procedure, neither the (N,E) nor the (X,Y) system defined in each GNSS station is aligned to the pendulum reference frame. Therefore, in order to compare the displacements from GNSS (X,Y)_i=1,2,3_ with those from pendulum, we have to align the pendulum frames to the GNSS (X,Y)_i=1,2,3_ frames.

Pendulum data are framed in a local reference system depending on the orientation of the ashlar. Generally, the reading table is mounted so that the two axes T-L are aligned with the Transversal and Longitudinal directions of the ashlar, which are assumed to be parallel to the upstream-downstream direction and to the dam crest direction, respectively. 

**Figure 5:**
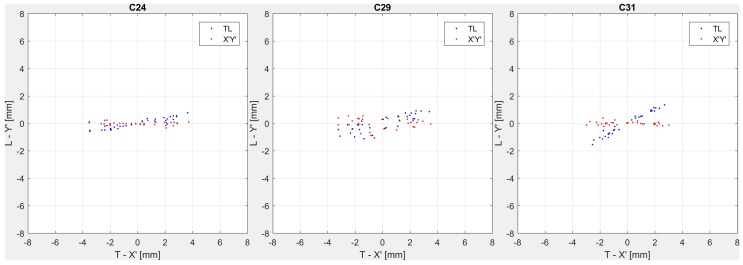
Displacements observed on ashlars 24 (**left**), 29 (**center**), 31 (**right**) by pendulums in the T-L (blue dots) and X′-Y′ (red dots) reference systems from 10 December 2014 to 9 December 2015.

The (T-L) pendulum displacements in the 24, 29 and 31 ashlars are plotted in Figure 5 (blue dots). Also, in the case of pendulum data, the displacements highlight different behaviors in the different ashlars, so that the rotation between each T-L frame and the corresponding (X′,Y′) frame is different. Thus, again, it must be underlined that three different reference systems must be assumed also for the three pendulums. In order to align the (T,L)_i=1,2,3_ axes to the cross-crest and along-crest directions, a procedure similar to the one devised for the GNSS coordinates has been adopted. 

The resulting rotation angles are 10.2°, 14.8° and 29.1° for ashlars 24, 29 and 31 respectively (with standard deviation of 0.7°, 4.5° and 2.0°). These angles have been used to rotate the full datasets in the (X′,Y′) frames. The results of this computation are represented in Figure 5 (red dots).

By adopting this procedure, we assume that the observed pendulum displacements and the observed GNSS displacements refer to reference systems which are pairwise parallel, that is we can consider (X,Y)_i_ parallel to (X′,Y′)_i_, i = 1, 2, 3.

**Figure 6:**
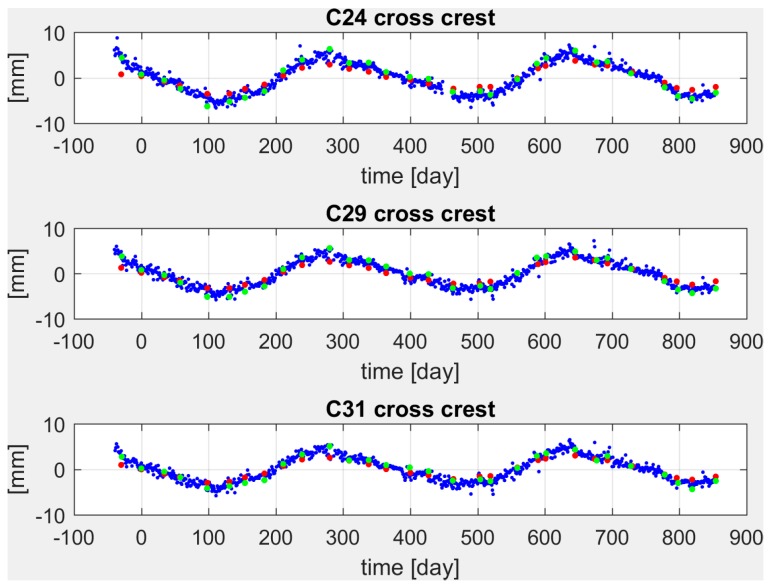
Displacements estimated on ashlars 24 (**top**), 29 (**center**), 31 (**bottom**) by pendulums (red dots) and GNSS (blue dots) in the cross-crest direction (green dots represent the collimator observations) from October 2014 to April 2017.

**Figure 7:**
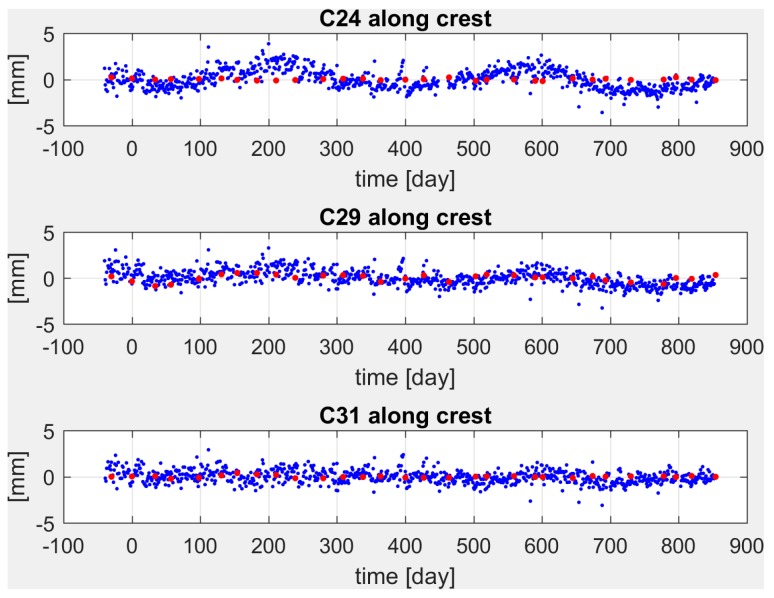
Displacements observed on ashlars 24 (**top**), 29 (**center**), 31 (**bottom**) by pendulums (red dots) and GNSS (blue dots) in the along-crest direction from October 2014 to April 2017.

In Figure 6 and Figure 7, the rotated pendulum and GNSS displacements are plotted for ashlars 24, 29, 31 in the cross-crest (X and X′) and along-crest (Y and Y′) directions respectively for the period October 2014 to April 2017. Time axis has been shifted so to have t = 0 on 10 December 2014, according to the zero set applied to the pendulums and collimator data.

In the cross-crest direction (Figure 6), the annual periodicity is clearly visible both in pendulums and GNSS displacements. Pendulum and GNSS data are in phase, describing the same oscillations although with slightly different amplitudes. This could be due to the propagation of pendulums data from the height of the measuring chamber (at 95 m a.s.l.) to the dam crest (at 120 m a.s.l.). In fact, a simple linear propagation was applied to the pendulum-observed displacements, which is likely to describe the ashlar deformation too roughly. For the along-crest direction the pendulum displacements are negligible, while GNSS detects displacements in the range from 5 mm for ashlar 24 to 4 mm for ashlar 29 and to the noise level when considering ashlar 31 (see Figure 7). Such small displacements detected by the pendulums could be explained again in terms of the inadequate transfer function that has been used in propagating the measurements, coupled with ashlars positions along the dam structure.

The previous outcomes highlight the specific behaviors of the monitoring techniques here presented. Nominally, GNSS monitoring systems are less accurate than the pendulum measurements but they allow describing the dam displacements with denser observations without losing the capability of detecting periodicity that is typical for this kind of monitoring problem. The higher frequency in the measurement acquisition leads to a better estimate of an analytical model able to properly describe (and predict) the expected displacements.

### 3.2. Analytical Modeling of Observed Dam Displacements

Different models are known in literature for modelling dam displacements [22,23,24]. We can distinguish two main methods: one based on physically observed quantities, such as water and air temperatures (they are referred to as ”deterministic” models) and one dependent by auxiliary variables as the time t (we will call this method as ”predictive” from now on). Both methods have advantages and disadvantages. Models adopting physical parameters directly express the relationship between mechanical properties and dam deformation. However, a reliable and accurate deterministic model is difficult to develop because of the uncertainty and complexity of the dam structure. On the other hand, predictive models completely disregard the dam structure design thus describing the deformation in a purely phenomenological way, although leading to relevant results. This second group of models are simpler and computationally straightforward, and allow predicting the future dam behavior. 

We decided to model the pendulum and the GNSS displacements using the predictive model by [25], where a formulation for describing deformation of buttress gravity dams has been proposed.

Following this approach, we modeled the crest displacement Δ*s* by a linear trend and two periodic components accounting for annual and semi-annual signals. 

Thus, such a model has the following expression:

(1) $$\Delta s=a+b\cdot t+c\cdot \cos\left(\frac{2\pi }{T_{1}}t\right)+d\cdot \sin\left(\frac{2\pi }{T_{1}}t\right)+e\cdot \cos\left(\frac{2\pi }{T_{2}}t\right)+f\cdot \sin\left(\frac{2\pi }{T_{2}}t\right)$$

where *t* is the time expressed in days, *T*_1_ is the annual period (365 days), *T*_2_ is the semi-annual period (*T*_2_ = *T*_1_/2) and a, b c, d, e and f are the coefficients to be estimated.

The linear term (coefficients a and b) describes potential plastic deformation whereas the harmonic annual/semi-annual components (coefficients c, d, e and f) describe periodic oscillations of the dam crest primarily due to the thermal effects caused by the differences between air and water temperature over the year (the reservoir temperature is lower than the temperature of the air in summer so that the dam moves along the upstream direction, the opposite occurring during winter).

For both GNSS and pendulum data of the three ashlars, model coefficients have been estimated by LSA of the observations in both the cross and along-crest directions. In order to tune the models, a *t-*test for null hypothesis has been applied on each estimated coefficient, with a 5% significance level. 

**Figure 8:**
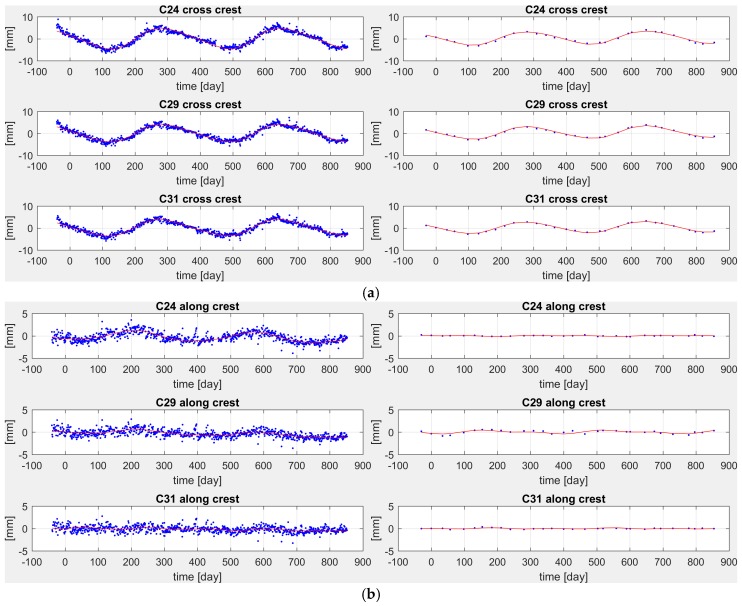
(**a**) Estimated models (red line) and observed displacements (blue dots) in the cross-crest direction by GNSS (**left**) and pendulums (**right**). (**b**) Estimated models (red line) and observed displacements (blue dots) in the along-crest direction by GNSS (**left**) and pendulums (**right**).

The estimated models are plotted in Figure 8a (cross-crest direction) and Figure 8b (along-crest direction).

**Figure 9:**
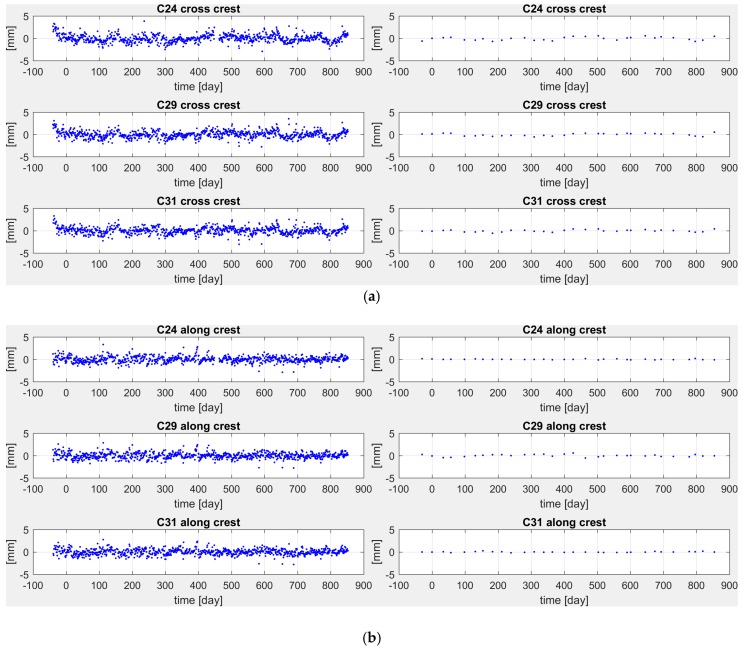
(**a**) Residuals of estimated models in the cross-crest direction by GNSS (**left**) and pendulums (**right**). (**b**) Residuals of the estimated models in the along-crest direction by GNSS (**left**) and pendulums (**right**).

The residual to estimated models are respectively plotted in Figure 9a,b and the statistics of these residuals are summarized in Table 1.

The discrepancies between modeled displacements confirm the underestimation of the displacements in the pendulum w.r.t. GNSS. The statistics of the residuals (see Table 1) show that GNSS is less precise than pendulum, which is quite expected. Pendulum residuals have a mean standard deviation of 0.22 mm, while GNSS residuals mean standard deviation is 0.75 mm. Nevertheless, this proves that GNSS (as well the pendulums) is able to monitor displacements such those described by the computed models that are of the order of some millimeters (see Figure 8a,b where the model displacements are given).

In this comparison among different techniques, collimator data can be also considered. As one can see in Figure 6, they are quite in line with the GNSS time series. The collimator data are by definition in the cross-crest direction and thus consistent with the rotated GNSS data. We also underline that the amplitude of the displacements is closer to the GNSS than to the one detected by pendulums. In our opinion, this enforces the hypothesis of an inadequate transfer function used for propagating the pendulum displacements to the dam crest.

Furthermore, residuals on collimator observations, once model (1) is fitted in these data, have a mean standard deviation of 0.81 mm. Thus, this is the less precise method for monitoring the dam displacements, which could be effectively substituted by GNSS-based techniques.

## 4. Comments and Conclusions

The comparative analyses that have been carried out on pendulum, GNSS and collimator data have shown that pendulum is the most accurate method for estimating the dam displacements. However, this method can be applied only in fixed and pre-defined points in the dam structure and thus an overall description of this is not possible. Furthermore, the pendulum data are collected inside the dam structure and cannot account properly for crest displacements, which are underestimated as we have shown in our investigations. 

The results obtained in this paper proved that GNSS techniques can be profitably used for a precise monitoring of the dam. Although less precise than pendulum, they are accurate enough. Also, GNSS instruments can be deployed on the dam crest and in a larger number than pendulums. This is particularly true nowadays, since low cost receivers can provide high precision positioning [26]. Also, GNSS data can be collected in highly automated way and time series can be estimated straightforwardly.

Furthermore, based on a set of data that was available, one can say that collimator data are in a quite good agreement with the GNSS-estimated displacements, even if less precise. However, although one can automate also this technique, this is quite expensive and, most of all, it can suffer for lateral refraction, which can give biased results. Therefore, collimator could be replaced by GNSS in view to have a more effective monitoring of the dam displacements.

Thus, in the end, we can state that a modern and efficient method for monitoring the displacements in a dam is to integrate some pendulum measurements with a number of GNSS devices placed on the dam crest. In such a way, GNSS-observed displacements can be compared and validated with pendulum ones on ashlars where both are present. The displacements analysis can be then densified in other points using GNSS so to have a detailed description of the dam deformation, thus ensuring a reliable estimate of possible anomalous behavior.

This setting can be further improved using ground-based SAR and, when available, total station observations data to complement GNSS and pendulum. This can be considered one possible research line for future investigations, which will also have a relevant impact on the analytical models to be used for integrating the observations coming from the different sensors.

## Figures and Tables

**Table 1:** Standard deviation of the differences between the observations and the corresponding models.

Ashlar	Sensor	Along-Crest Stand. Dev. (mm)	Cross-Crest Stand. Dev. (mm)
no. 24	pendulum	0.08	0.37
	GNSS	0.69	0.88
	collimator	--	0.84
no. 29	pendulum	0.26	0.28
	GNSS	0.68	0.82
	collimator	--	0.83
no. 31	pendulum	0.10	0.23
	GNSS	0.67	0.77
	collimator	--	0.78

## References

[1] Scaioni M., Marsella M., Crosetto M., Tornatore V., Wang J. (2018). Sensors for Dam Deformation Monitoring.

[2] Antova G. 3D Laser Scanning for Dam Deformation Monitoring. Proceedings of the 7th International Scientific Conference-SGEM.

[3] Alba M., Fregonese L., Prandi F., Scaioni M., Valgoi P. (2006). Structural monitoring of a large dam by terrestrial laser scanning. Int. Arch. Photogramm. Remote Sens. Spat. Inf. Sci..

[4] Di Martire D., Iglesias R., Monells D., Centolanza G., Sica S., Ramondini M., Pagano L., Mallorquí J.J., Calcaterra D. (2014). Comparison between differential SAR interferometry and ground measurements data in the displacement monitoring of the earth-dam of Conza della Campania (Italy). Remote Sens. Environ..

[5] Honda K., Nakanishi T., Haraguchi M., Mushiake N., Iwasaki T., Satoh H., Kobori T., Yamaguchi Y. Application of exterior deformation monitoring of dams by DInSAR analysis using ALOS PALSAR. Proceedings of the 2012 IEEE International Geoscience and Remote Sensing Symposium (IGARSS).

[6] Anghel A., Vasile G., Boudon R., d’Urso G., Girard A., Boldo D., Bost V. (2016). Combining spaceborne SAR images with 3D point clouds for infrastructure monitoring applications. ISPRS J. Photogramm. Remote Sens..

[7] Milillo P., Perissin D., Salzer J.T., Lundgren P., Lacava G., Milillo G., Serio C. (2016). Monitoring dam structural health from space: Insights from novel InSAR techniques and multi-parametric modeling applied to the Pertusillo dam Basilicata, Italy. Int. J. Appl. Earth Obs. Geoinf..

[8] Mascolo L., Nico G., Di Pasquale A., Pitullo A. (2014). Use of advanced SAR monitoring techniques for the assessment of the behaviour of old embankment dams. Int. Soc. Opt. Photonics.

[9] De Loach S.R. (1989). Continuous deformation monitoring with GPS. J. Surv. Eng..

[10] Behr J., Hudnut K., King N. Monitoring structural deformation at Pacoima dam, California using continuous GPS. Proceedings of the 11th International Technical Meeting of the Satellite Division of the Institute of Navigation.

[11] Rutledge D.R., Meyerholtz S.Z., Brown N., Baldwin C. (2006). Dam stability: Assessing the performance of a GPS monitoring system. GPS World.

[12] Chrzanowski A., Szostak-Chrzanowski A. (2009). Deformation monitoring surveys-old problems and new solutions. Rep. Geodesy.

[13] Drummond P. Combining CORS Networks, Automated Observations and Processing, for Network Rtk Integrity Analysis and Deformation Monitoring. Proceedings of the 15th FIG Congress Facing the Challenges.

[14] Van Cranenbroeck J. State of the Art in Structural Geodetic Monitoring Solutions for Hydro Power Dams. Proceedings of the FIG Working Week.

[15] Kaftan V.I., Ustinov A.V. (2013). Use of global navigation satellite systems for monitoring deformations of water-development works. Power Technol. Eng..

[16] Dardanelli G., La Loggia G., Perfetti N., Capodici F., Puccio L., Maltese A. (2014). Monitoring displacements of an earthen dam using GNSS and remote sensing. SPIE Remote Sens..

[17] Yavaşoğlu H.H., Kalkan Y., Tiryakioğlu İ., Yigit C.O., Özbey V., Alkan M.N., Bilgi S., Alkan R.M. (2018). Monitoring the deformation and strain analysis on the Ataturk Dam, Turkey. Geomat. Nat. Hazards Risk.

[18] Pipitone C., Maltese A., Dardanelli G., Lo Brutto M., Loggia G.L. (2018). Monitoring water surface and level of a reservoir using different remote sensing approaches and comparison with dam displacements evaluated via GNSS. Remote Sens..

[19] Radhakrishnan N. (2014). Application of GPS in structural deformation monitoring: A case study on Koyna dam. J. Geomat..

[20] Barzaghi R., Pinto L., Monaci R. The monitoring of gravity dams: Two tests in Sardinia, Italy. Proceedings of the FIG Working Week 2012.

[21] Galán-Martín D., Marchamalo-Sacristán M., Martinez-Marín R., Sánchez-Sobrino J.A. (2010). Geomatics applied to dam safety DGPS real time monitoring. Int. J. Civil Eng..

[22] Dai W., Huang D., Liu B. (2015). A phase space reconstruction based single channel ICA algorithm and its application in dam deformation analysis. Surv. Rev..

[23] Montillet J.P., Szeliga W.M., Melbourne T.I., Flake R.M., Schrock G. (2016). Critical Infrastructure Monitoring with Global Navigation Satellite Systems. J. Surv. Eng..

[24] Alevizakou E.G., Pantazis G. (2017). A comparative evaluation of various models for prediction of displacements. Appl. Geomat..

[25] De Sortis A., Paoliani P. (2007). Statistical analysis and structural identification in concrete dam monitoring. Eng. Struct..

[26] Caldera S., Realini E., Barzaghi R., Reguzzoni M., Sansò F. (2016). Experimental study on low-cost satellite-based geodetic monitoring over short baselines. J. Surv. Eng..

